# Adhesion molecules of detrusor muscle cells are influenced by a hypercholesterolemic diet or bladder outlet obstruction in a wistar rat model

**DOI:** 10.1186/1471-2490-13-50

**Published:** 2013-10-20

**Authors:** José Pontes-Júnior, Ricardo Luís Vita Nunes, Sabrina Thalita dos Reis, Luiz Carlos N de Oliveira, Nayara Viana, Katia Ramos Moreira Leite, Homero Bruschini, Miguel Srougi

**Affiliations:** 1 Laboratory of Medical Investigation – LIM 55, Urology Department, University of São Paulo Medical School, São Paulo, Brazil; 2 Universidade Nove de Julho, São Paulo, Brazil

**Keywords:** Cell adhesion molecules, Bladder dysfunction, Hypercholesterolemic diet, Bladder outlet obstruction

## Abstract

**Background:**

Cell adhesion molecules (CAMs) are essential for maintaining tissue integrity by regulating intercellular and cell to extracellular matrix interactions. Cadherins and catenins are CAMs that are located on the cell membrane and are important for adherens junction (AJ) function. This study aims to verify if hypercholesterolemic diet (HCD) or bladder outlet obstruction (BOO) promotes structural bladder wall modifications specific to alterations in the expression of cadherins and catenins in detrusor muscle cells.

**Methods:**

Forty-five 4-week-old female Wistar rats were divided into the following three groups: group 1 was a control group that was fed a normal diet (ND); group 2 was the BOO model and was fed a ND; and group 3 was a control group that was fed a HCD (1.25% cholesterol). Initially, serum cholesterol, LDL cholesterol and body weight were determined. Four weeks later, groups 1 and 3 underwent a sham operation; whereas group 2 underwent a partial BOO procedure that included a suture tied around the urethra. Six weeks later, all rats had their bladders removed, and previous exams were repeated. The expression levels of N-, P-, and E-cadherin, cadherin-11 and alpha-, beta- and gamma-catenins were evaluated by immunohistochemistry with a semiquantitative analysis.

**Results:**

Wistar rats fed a HCD (group 3) exhibited a significant increase in LDL cholesterol levels (p=0.041) and body weight (p=0.017) when compared to both groups that were fed a normal diet in a ten-week period. We found higher β- and γ-catenin expression in groups 2 and 3 when compared to group 1 (p = 0.042 and p = 0.044, respectively). We also observed Cadherin-11 overexpression in group 3 when compared to groups 1 and 2 (p = 0.002).

**Conclusions:**

A HCD in Wistar rats promoted, in addition to higher body weight gain and increased serum LDL cholesterol levels, overexpression of β- and γ-catenin in the detrusor muscle cells. Similar finding was observed in the BOO group. Higher Cadherin-11 expression was observed only in the HCD-treated rats. These findings may be associated with bladder dysfunctions that occur under such situations.

## Background

Bladder dysfunction is a syndromic diagnosis with several causes and different clinical presentations. It usually causes symptoms that affect patient quality of life, can occur at any age in both sexes and is pathologically characterised by the existence of abnormal bladder contraction or relaxation. This condition is not uncommon; it was estimated that more than a third of the United Kingdom population older than 40 years have an overactive bladder diagnosis [1].

The pathophysiological mechanism of bladder dysfunction is not well understood; however bladder outlet obstruction (BOO), metabolic diseases, neurologic conditions, inflammation and aging probably have an important role. In addition, accumulating data in the literature indicates that structural and interaction changes between detrusor smooth muscle cells are important in bladder dysfunction pathogenesis. Supporting this concept, a dysfunctional pattern of muscle fibres that are caused by loss of intercellular adhesion has been demonstrated to be the morphological correlation of a hyperactive detrusor or an obstructed bladder [2,3]. BOO is a prevalent condition associated with bladder dysfunction and if not properly treated may lead to irreversible fibrosis in bladder wall [4].

Hypercholesterolemia is a risk factor in many cardiovascular diseases and may also affect the genitourinary tract. Corroborating this notion, Son et al. demonstrated correlation between hypercholesterolemia and detrusor overactivity in experimental model [5]. Our group evaluated the collagen composition of bladder wall in two rat models of BOO and hypercholesterolemia and found that both groups had the same morphological alterations regarding thin collagen fibers and the amounts of type III collagen expressed when compared to the control group; indicating that despite the different physiopathology, some molecular changes in BOO may be similar to that observed in metabolic diseases [6]. However, up to now the complete pathogenesis of bladder dysfunction secondary to metabolic disease and obstruction in humans is not defined.

The cellular basis of bladder contraction is not an independent mechanism but rather a result of a group of functional components of smooth muscle cells and their extracellular matrices that act cooperatively with myofibroblasts to contract or relax. Therefore, proper intercellular adhesion and communication, which are related to cell adhesion molecule (CAM) expression, are required for normal bladder function.

It is acknowledged that interaction between detrusor smooth muscle cells and myofibroblasts, coupled via gap junctions, is required for coordinated and synchronised detrusor contraction. However, the sole interaction promoted by gap junctions is insufficient to ensure an optimal contraction; stronger intercellular adhesions such as adherens junctions (AJs) are necessary to avoid breaking muscle bundles during contraction and to assure gap junction functioning. The protein composition of AJs is mainly comprised of CAMs, especially catenins and cadherins.

Cadherins are transmembrane calcium-dependent proteins that regulate homotypic intercellular adhesion. The intracellular domain is anchored to the cytoskeleton through a complex of alpha-, beta- and gamma-catenins, whereas the extracellular portion is connected with the extracellular domain of adjacent cell’s cadherin. The cadherin-catenin complex is essential for AJs; moreover, expression of the entire complex in detrusor muscle cells is necessary for proper contraction.

E-cadherin is commonly expressed in the AJ in epithelial tissue. Regarding the cadherin that is expressed in smooth muscle cell, the scarce literature indicates that cadherin-11 is the probable candidate [7]. Kuijpers et al. observed cadherin-11 expression in detrusor smooth muscle cells in biopsies from radical cystectomy specimens, suggesting that it is important for adhesion between detrusor smooth muscle cells and myofibroblasts, which is required for a coordinated bladder contraction [8].

If the cadherin-11-catenin complex is necessary for physical coupling of detrusor muscle cells and coordinated bladder contractions [8], one can theorise that pathological conditions that interfere with bladder homeostasis may be associated with disturbance of this complex, but to date the literature regarding CAM expression is insufficient to define their role in bladder dysfunction pathogenesis. We theorize that CAM expression changes in detrusor muscle cells may represent the molecular basis of bladder dysfunction. This study aims to verify if (HCD) or (BOO) promote structural bladder wall modifications specific to cadherin and catenin expression level changes in detrusor muscle cells.

## Methods

### Experimental protocol

We prospectively evaluated forty-five 4-week-old female Wistar rats that were divided into the following three groups consisting of 15 rats each: group 1 was a control group and was fed a normal diet (ND); group 2 was the BOO model and was fed a ND; and group 3 was a control group and was fed a HCD (1.25% cholesterol diet). The HCD model in rats employed in our study was earlier described by Nunes et. al. [6]. Initially, serum levels of cholesterol, LDL cholesterol and body weight were determined. Four weeks later, group 2 underwent a partial BOO procedure, whereas groups 1 and 3 underwent a similar sham operation.

The partial BOO procedure was also previously described [9]. Briefly, under general anaesthesia, the skin was shaved, prepped with an iodine/alcohol mixture and a low midline longitudinal incision was made. The urethra was circumferentially dissected and released from the surrounding periurethral tissue to enable the confection of the BOO procedure. In groups 1 and 3, the procedure was concluded at this time. In group 2, an unabsorbed 5–0 nylon suture was passed and tied loosely around the urethra with a 22G needle inside, leaving approximately 1 mm of residual urethral lumen [9].

All groups were still fed their particular diet (groups 1 and 2 with the normal diet and group 3 with the HCD). Six weeks later, for a total of ten weeks of study, cholesterol, LDL cholesterol and body weight were reassessed. Subsequently, all of the rats were euthanised in a CO2 chamber and had their bladders removed. We chose 6 weeks after the BOO procedure because this was the time necessary to cause changes in detrusor muscle cells secondary to the partial outlet obstruction [6,9]. An open full bladder wall thickness biopsy was performed to provide tissue for CAM expression analysis, and after fixation, the tissue was embedded in paraffin.

**Figure 1:**
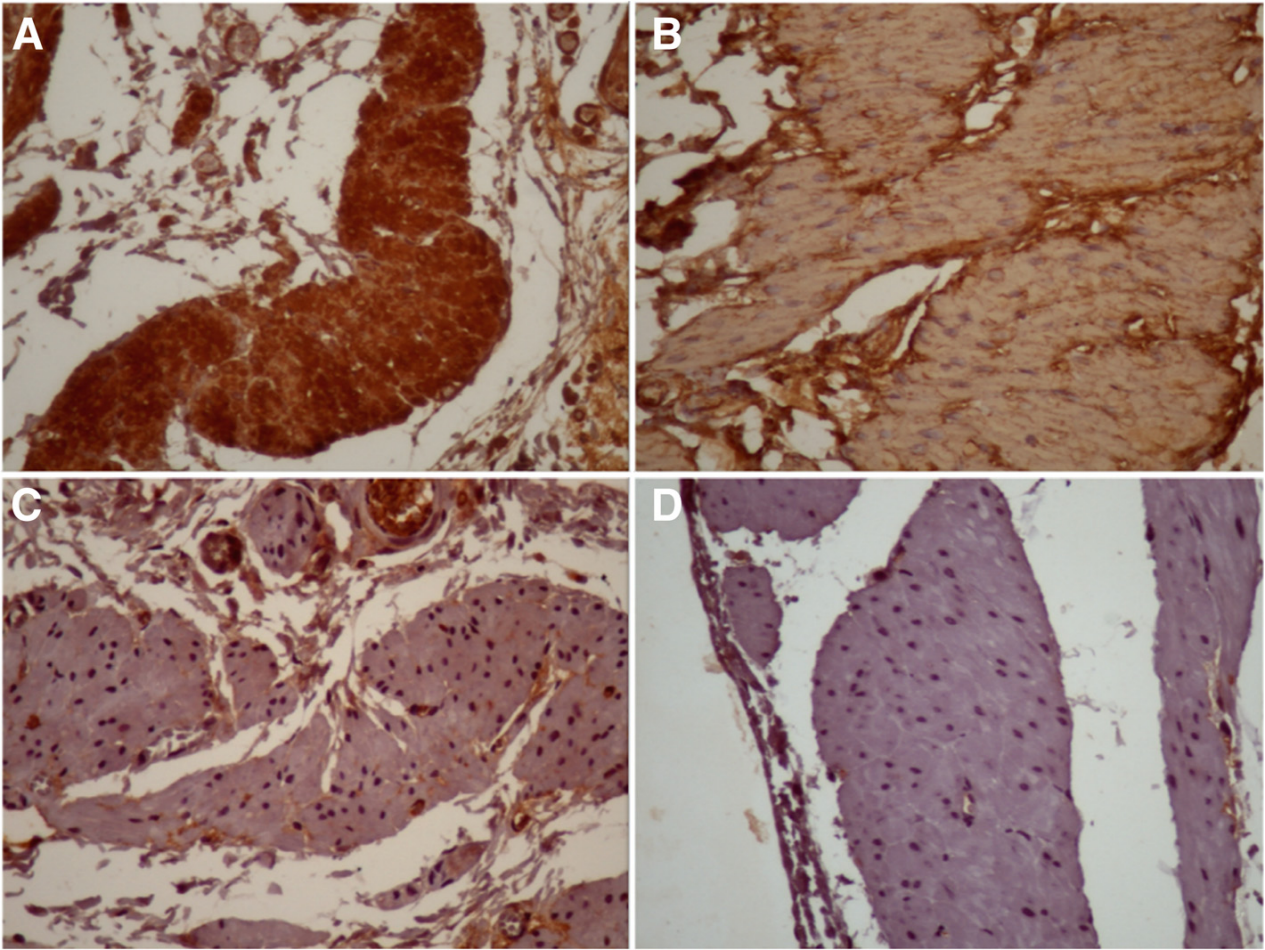
Examples of the four categories of the CAM expressions semiquantitative analysis’ semiquantitative analysis through immunohistochemistry used in the study: examples of absent (A), weak (B), intermediate (C) and strong (D) expression of cadherin-11 in detrusor muscle cells; magnification 400x.

Expression levels of N-, P-, and E-cadherin; cadherin-11; and α-, β- and γ-catenins were evaluated through immunohistochemistry by the same uropathologist, (KRML), and semiquantitative analyses were performed for all of the antibodies. The expression was divided into four groups according to the staining intensity as follows: 0 representing negative staining, 1 representing weak staining, 2 representing intermediate staining and 3 representing strong staining. In Figure 1, we present examples of the semiquantitative analysis employed in the study.

It was previously demonstrated that normal epithelial and mesenchymal cells display diffusely strong expression of the Cadherin-catenin complex molecules at cell membrane or cytoplasm [10,11]. For that reason and as described by others [11], in our study the CAM expression was considered as higher when the staining was strong and diffusely present in more than 70% of the detrusor muscle cells evaluated and lower when the staining was below that value.

### Immunohistochemistry

Histological examinations of the specimens were performed in formalin-fixed and paraffin-embedded sections that were stained with haematoxylin and eosin. The samples underwent a heat-induced antigen retrieval process using citrate buffer (1 mM, pH 6.0). The slides were incubated overnight at 4°C with a specific monoclonal antibody. The LSAB system was used for the immunostaining (Dako Cytomation, CA). Colour was developed through a reaction with a 3,3′-diaminobenzidine substrate-chromogen solution followed by counterstaining with Harris haematoxylin. The slides were dehydrated, coverslipped and observed under a light microscope. The specific antibodies and their respective dilutions are listed in Table 1.

**Table 1:** Antibodies and their respective dilutions

**Antibody**	**Manufacturer**	**Dilution**
E-Cadherin	Dako, CA, USA	1:50
β-Catenin	BD, NJ, USA	1:50
γ-Catenin	Zymed, CA, USA	1:100
α-Catenin	Santa Cruz, CA, USA	1:100
Cadherin-11	Invitrogen, CA, USA	1:50
N-Cadherin	Dako, CA, USA	1:50
P-Cadherin	Abcam, Cambridge, UK	1:20

Statistical analyses were performed using SPSS version 12.0 software for Windows. The metabolic changes were evaluated through the ANOVA model with repeated measure. We compared CAM expressions between the three groups with the generalisation of Fisher’s exact test and chi-square test; post hoc analysis with Bonferroni correction was used to test the differences between each group and p < 0.05 was considered statistically significant. Approval for the study was given by the Institutional Board of Ethics of our (CAPPesq - Comissão de Ética para Análise de Projetos de Pesquisa do HCFMUSP n°1074/04). The animals received humane care in compliance with the Guide for care and use of laboratory animals (NIH Publication 85–23, revised 1985).

## Results

One rat from group 2 developed urinary retention due to excessive tension applied during the BOO procedure and was excluded from the study, the remaining 44 rats completed the protocol for CAM expression analysis.

To compare the metabolic changes according to diet type, the ND-treated rats in groups 1 and 2 were combined and compared with the HCD-treated group (group 3). Twelve rats from groups 1 and 2 were excluded from this analysis because of loss of data due to storage problems. All biochemical and weight gain parameters are summarised in Table 2. From the start point we see that HCD group shows the same total cholesterol, LDL levels and body weight than controls, the p-values observed were 0.462, 0.368 and 0.196, respectively.

**Table 2:** Total cholesterol, LDL cholesterol and body weight values in 32 Wistar rats stratified by diet type

	**Time**		
	**t = 0**	**10 weeks**	**p-value**
**Total cholesterol (mg/mL)**			
**Normal diet**	80.9 ± 3.8	87.6 ± 2.1	0.288
**HC diet**	85.7 ± 6.3	91.0 ± 5.5	0.427
p-value	0.462	0.376	
**LDL cholesterol (mg/mL)**			
**Normal diet**	48.4 ± 2.8	48.2 ± 2.1	0.964
**HC diet**	53.7 ± 5.8	70.9 ± 5.3	0.007
p-value	0.368	0.041	
**Body weight (g)**			
**Normal diet**	83.2 ± 3.7	239.8 ± 7.2	<0.001
**HC diet**	94.5 ± 4.3	281.5 ± 7.8	<0.001
p-value	0.196	0.017	

The data were expressed by mean ± standard error.

After the ten-week study, the HCD cases exhibited a significant increase of LDL cholesterol levels and body weight when compared to rats fed a ND (groups 1+2) (p=0.041). The average weight gain in HCD group was 187.0 ± 8.2 g compared to 156.6± 8.2 g in controls (p=0.017). No difference was found in the serum total cholesterol levels between the rats fed and HCD or ND.

For CAM expression analysis, all groups were compared independently, and all rats, excluding 1 from group 2, were considered. The expressions of N-, P-, and E-cadherin and cadherin-11 were detected in cytoplasmic cell membrane, whereas alpha-, beta- and gamma-catenins were expressed in both the cytoplasm and cell membrane. The number and percentage of cases with higher expression for each CAM is depicted in Table 3.

**Table 3:** Number and percentage of cases with higher CAM expression values in 45 female Wistar rats are stratified as follows: group 1, control fed a ND; group 2, BOO-treated model fed a ND and group 3) control fed a HCD

	**Group 1**	**Group 2**	**Group 3**	
**CAM**	**Control**	**BOO**	**HCD**	**p value**
	**N° (%)**	**N° (%)**	**N° (%)**	
E-Cadherin	6 (40.0%)	6 (42.9%)	5 (33.3%)	0.393^1^
N-Cadherin	0	3 (21.4%)	5 (33.3%)	0.056^2^
P-Cadherin	1 (6.7%)	0	4 (26.6%)	0.169^2^
Cadherin-11	3 (20.0%)	5 (35.7%)	12 (80.0%)	0.003^1^
β-Catenin	2 (20.0%)	8 (66.7%)	5 (71.4%)	0.049^2^
α-Catenin	4 (40.0%)	7 (58.3%)	3 (42.9%)	0.717^2^
γ-Catenin	1 (10.0%)	6 (50.0%)	4 (57.1%)	0.084^2^

^1^Chi-square test ^2^Generalisation of Fisher’s exact.

The global comparison of Cadherin-11 expression among the 3 groups was statistically significant (p=0.003). At post-hoc analysis we found that the difference was due the higher Cadherin-11 expression in HCD group when compared to control and BOO groups (p=0,002). Cadherin-11 expression was also higher in the BOO group when compared with group 1 (35 vs. 20%); however the difference was not significant (p=0.4270).

The β-catenin expression in global comparison was also statistically significant (p=0.049). At post-hoc analysis we observed that HCD and BOO groups were similar (p>0.999). The β-catenin expression was higher in HCD and BOO groups when compared to controls (p=0.042).

The γ-catenin expression among the 3 groups only reaches marginally significance (p=0.084). The expression was similar between HCD and BOO groups (p>0.999); however γ-catenin expression was significatively higher in BOO and HCD groups when compared to controls (p=0.044). We did not find any differences in expression amongst the groups for the following CAMs: E-cadherin, N-cadherin, P-cadherin or alpha-catenin.

## Discussion

This is the first study in the literature to demonstrate that a HCD or the presence of BOO in Wistar rats was associated with increased beta- and gamma-catenins expression levels in detrusor smooth muscle cells. We also observed increased cadherin-11 expression in HCD group when compared to BOO and control groups. We hypothesise that these pathological conditions cause detrusor homeostasis disruption and that the molecular response to this condition is reflected by altered CAM expression in order to improve the mechanically coupling of muscle cells and detrusor contraction.

In normal epithelial tissues, mechanical cell coupling is dependent on an AJ, which mediates the adhesion of cells to their neighbours or to the extracellular matrix. The AJs are located at the plasma membrane and are mainly formed by CAMs that anchor cytoskeletal myofilaments to the cell membrane [12,13]. It has been demonstrated that the AJ is also the predominant structure responsible for detrusor smooth muscle cell coupling. Carey et al. determined immunohistochemically that the entire cell membrane of normal detrusor smooth muscle cells exhibited the presence of AJs [14]. If AJs are essential for mechanical detrusor muscle coupling in physiological conditions, a similar importance was also demonstrated for them in dysfunctional bladders [15].

Smooth muscle cells in the bladder wall are mechanosensitive, which enables them to respond to the mechanical stretch stress that occurs after BOO. The smooth muscle cells undergo modifications of gene expression and protein synthesis that cause a hypertrophic state that is necessary to increase intravesical pressure, overcome BOO and result in micturition. Although the physiopathological mechanism whereby BOO causes urinary symptoms is not clear, several morphologic and functional modifications of the bladder detrusor muscle cells have been described in BOO patients that could play a direct role in determining voiding dysfunction and symptoms [16]. Because cells must be extensively connected both electrically and structurally to produce coordinated contractions [17], we theorise that the modifications observed n BOO patients may be related, in part, to changes in CAM expression.

Cadherin-11, also known as osteoblast-cadherin, belongs to the type 2 cadherin subgroup and is a marker of the connected cells of the mesenchyme. We supposed that the molecular change necessary to increase smooth muscle contractions in BOO cases would be the higher Cadherin-11 expression. Corroborating the role of cadherin-11 in bladder dysfunction, Roosen et al. evaluated CAM expression using immunohistochemistry in 32 patients with DO and observed co-localisation of cadherin-11 and beta-catenin in detrusor and myofibroblast cells and upregulation of cadherin-11 in the suburothelium [18]. Roosen et al. concluded that these CAM might have a significant role in overactive bladder pathogenesis [18].

The same mechanism may be postulated for HCD-treated rats, as higher cadherin-11 and catenins levels were also found in this group. The observation of higher cadherin-11 expression in 80% of HCD rats indicates that this is the cadherin that maintain the cadherin-catenin complex in detrusor. If one acknowledges that HCD is associated with a loss of detrusor muscle cells, increases in fibroblastic cells and collagen deposition due chronic ischemia, the higher expression of proteins of the cadherin-catenin complex in such condition would represent a compensatory effort to recover muscle contraction. In a previous report, using the same methodology employed in the present article, we demonstrated that BOO and HCD groups exhibited similar higher expression of thin collagen fibers and type III collagen when compared to controls, indicating that, despite the different physiopathology, both conditions share some molecular changes at bladder wall [6]. Supporting this hypothesis, Nomiya et al. also demonstrated increased bladder activity characterized by higher reflex bladder contraction in a rat model of atherosclerosis-induced chronic bladder ischemia [19].

In our study, Cadherin-11, beta- and gamma-catenins expression were higher in BOO group when compared to controls, however it reached significance only for the beta and gamma catenins. This molecular finding may enable better contraction by improving communication between smooth muscle cells and myofibroblasts and by increasing adhesion between detrusor muscle cells, as these catenins play a pivotal role in AJ that is critical for cell-cell coupling.

The three catenins connect and anchor the cadherin at the cell membrane to the cytoskeletal actin and are essential for cell-cell adhesion. A loss of catenin, despite normal cadherin expression, compromises the adhesion complex. Supporting this concept, it has been demonstrated that expression of alpha- and beta-catenin, in addition to cadherin, is required for adequate cardiac muscle cell contraction in rats [20]. In our study, we observed significant increases in beta- and gamma-catenin levels in the BOO- and HCD-treated groups compared with the control group. The increased levels of almost all proteins of the cadherin-catenin complex in detrusor muscle cells of BOO- or HCD-treated rats may indicate a tendency to preserve cell adhesion that is essential for smooth cell contraction.

The role of CAM in bladder dysfunction is also emphasised by studies in geriatric patients in which reductions of AJ were commonly observed in DO and BOO patients. Elbadawi et al. evaluated 35 elderly patients with bladder dysfunction that was characterised by DO, and by employing electron microscopy, they found a distinctive disjunction structural pattern that was characterised by moderately widened intercellular spaces and scarce intermediate muscle cell junctions [21]. Other studies on the geriatric population affected by BOO or DO also found reductions of junctions between detrusor muscle cells [22,23]. We believe that these histological dysfunctional findings reflect the phenotype associated with changes in CAM expression.

Kuijipers et al. evaluated CAM expression through immunofluorescence in bladder biopsies from eight radical cystectomy specimens and found an absence of E-cadherin and gamma- and alpha-catenins and the presence of cadherin-11 and beta-catenin in detrusor muscle cells [8]. In accordance with our conclusion, Kuijipers et al. postulated that cadherin-11 may have an important role in normal bladder contraction by connecting smooth detrusor muscle cells and myofibroblasts, which is necessary for normal bladder contraction.

In our series, we also observed reduced E-cadherin expression in the majority of the cases regardless the group; this finding is expected because this CAM is usually expressed in epithelial tissues. The difference between Kuijipers’ analysis and our study concerning alpha- and gamma-catenins expression levels may be explained by the different tissues evaluated in these series. The former study evaluated normal bladder tissue adjacent to bladder cancer, whereas ours focused on normal rat bladder tissue.

A previous study published by Wagener et al., in contrast to ours, reported that no cadherin or catenin expression levels were observed by immunohistochemistry in the detrusor muscle cells of DO and BOO patients, and concluded that such CAMs are not involved in smooth muscle cell adhesion in these pathological conditions [23]. This disparity may be explained by the employment of a polyclonal pan-cadherin antibody, whereas in our analysis, we used monoclonal specific antibodies against N-, P-, and E-cadherin and cadherin-11. The time of CAM expression analyses is another difference between the protocols; our results reflect the changes that occur at 10 weeks in rats, whereas the former study evaluated chronic established pathological conditions in human patients. CAM expression alterations in detrusor muscle cells may represent an early finding that is not captured when only chronic cases are evaluated.

A limitation of our data is that we evaluated bladders from rats; therefore, analysis of CAM expression in human bladders of BOO and dyslipidemic patients is necessary to ensure that these findings are applicable to dysfunctional human bladder. In addition, the results of the present study should be regarded as hypothesis generating; therefore, confirmatory studies employing quantitative methods of protein expression such as western blotting or gene expression analysis through RT-PCR are necessary to confirm our results.

## Conclusions

A hypercholesterolemic diet in Wistar rats promoted, in addition to higher body weight gain and an increased serum LDL cholesterol level, the overexpression of β- and γ-catenin in detrusor muscle cells, similar finding was observed in the BOO group. Cadherin-11 overexpression was observed only in the HCD-treated rats. These findings may be associated with bladder dysfunctions that occur under such situations.

## Abbreviations

CAM: Cell adhesion molecule; HCD: Hypercholesterolemic diet; BOO: Bladder outlet obstruction; AJ: Adherens junction; DO: Detrusor overactivity; ND: Normal diet; ANOVA: Analysis of variance.

## Competing interests

The authors have no conflicts of interest to declare.

## Authors’ contribution

JPJ principal investigator, collaboration in the experiment and manuscript. RLVN First co-author and participation in the experiment. STR assistance with the animal model set up and experiment. LCNO carried out the experiment and animal model assistance. NV carried out experiment and Immunohistochemistry. KRML carried out the cell adhesion molecules expression evaluation. HB conceived the study and participation in paper writing. MS project elaboration and assistance with the results’ analysis. All authors read and approved the final manuscript.

## Pre-publication history

The pre-publication history for this paper can be accessed here:

http://www.biomedcentral.com/1471-2490/13/50/prepub
